# Linking Children’s Emotional Experiences of Space with Health-Oriented Urban Design: Towards School Streets in Belgrade

**DOI:** 10.3390/ijerph23040516

**Published:** 2026-04-17

**Authors:** Milena Vukmirović

**Affiliations:** Faculty of Forestry, University of Belgrade,1 Kneza Višeslava, 11000 Belgrade, Serbia; milena.vukmirovic@sfb.bg.ac.rs; Tel.: +381-63603378

**Keywords:** children, school streets, urban design, public space, health, active mobility, participatory methods, emotional mapping

## Abstract

**Highlights:**

**Public health relevance—How does this work relate to a public health issue?**

- Everyday school routes are emotionally significant environments that shape children’s sense of safety, comfort, and willingness to move actively.
- Children’s route experiences reveal how traffic, legibility, and environmental quality are linked to everyday feelings of stress or emotional ease.

**Public health significance—Why is this work of significance to public health?**

- Children’s participatory evidence revealed recurring stress nodes associated with traffic exposure, pedestrian discontinuity, and spatial insecurity along everyday school routes.
- Positive route experiences were associated with greenery, legibility, and calmer street environments, suggesting their relevance to emotional regulation and well-being.

**Public health implications—What are the key implications or messages for practitioners, policy makers and/or researchers in public health?**

- A health-oriented interpretation of children’s emotional-spatial experience can strengthen the assessment of school-route environments beyond conventional traffic metrics.
- The findings support context-sensitive School Street interventions in Belgrade, particularly through traffic-calming measures, safer crossings, improved pedestrian continuity, and increased greenery.

**Abstract:**

Children’s everyday routes to school are increasingly recognised as important environments shaping physical and mental well-being. Yet, their emotional dimension remains insufficiently integrated into health-oriented urban design research, particularly in cities without formalised School Street policies. This study examines how children in Belgrade perceive and emotionally experience their everyday school routes and how such evidence can inform context-sensitive urban design. A mixed-method, child-centred participatory approach was applied with primary school pupils, combining participatory evaluation boards, cognitive route mapping, photo documentation, and facilitated classroom discussion. The material was analysed through qualitative coding, triangulation, and a health-oriented reinterpretation of the SCORELINE framework (h_SCORELINE). The findings reveal recurring stress nodes associated with traffic-dominated streets, complex crossings, obstructed sidewalks, and poorly legible route segments, which children linked to fear, discomfort, and insecurity. By contrast, greenery, recognisable landmarks, visually calm environments, and wider pedestrian spaces emerged as joy nodes associated with comfort, enjoyment, and emotional ease. These patterns suggest that children’s emotional-spatial evidence can enrich the assessment of school-route environments beyond conventional traffic indicators alone. By linking children’s lived experiences with health-oriented urban design, the study provides evidence-based support for the gradual introduction of School Streets in Belgrade. It offers a transferable framework for integrating child-centred experiential knowledge into healthier street design.

## 1. Introduction

Children’s everyday mobility environments are increasingly recognised as important determinants of physical, mental, and emotional well-being. Daily routes to school represent one of the most frequent and formative encounters between children and the built environment, shaping not only patterns of physical activity but also emotional states, perceptions of safety, comfort, autonomy, and belonging. A growing body of interdisciplinary research demonstrates that children’s emotional experiences of everyday environments—such as feelings of enjoyment, fear, stress, and perceived control—are closely linked to psychological well-being, life satisfaction, and health-related behaviours [1,2,3,4,5].

Positive emotional perceptions of school routes are consistently associated with higher levels of active mobility and greater psychological well-being. In contrast, negative emotional responses, such as fear, anxiety, or stress, can restrict independent movement and discourage walking or cycling, even over short distances [1,2,6,7]. These emotional responses emerge through children’s everyday encounters with traffic, infrastructure, social environments, and environmental quality. As such, school routes function not only as transport corridors but also as lived environments that shape everyday well-being.

### 1.1. Children’s Everyday Mobility, Emotional Experience, and Health

Recent literature emphasises that children’s emotional experiences of everyday environments play a central mediating role in the relationship between spatial conditions and health outcomes. Feelings of safety, enjoyment, and comfort support emotional regulation and psychological well-being, whereas repeated exposure to stress-inducing environments can contribute to anxiety, fatigue, and psychosomatic symptoms [2,8,9]. Therefore, emotional perceptions operate as mechanisms through which the built environment shapes children’s health-related behaviours and experiences.

Autonomy is a particularly important dimension of this relationship. Research indicates that autonomous mobility—understood as children’s perceived freedom and control over movement—is more strongly associated with well-being than independence alone [3,4,5]. Children who perceive their everyday environments as safe, legible, and supportive are more likely to walk or cycle independently, reinforcing both physical activity and positive emotional states [1,4,6].

The restorative qualities of everyday environments further shape these experiences. Access to greenery, visual openness, and calm sensory conditions along daily routes have been shown to reduce stress and support emotional comfort, even outside formal green spaces [7,10,11,12]. Conversely, exposure to noise, air pollution, thermal discomfort, and visually degraded environments is associated with increased stress and reduced well-being [9,10,13]. Taken together, these findings suggest that the everyday streetscape used by children is not a neutral backdrop to mobility, but an active component of their health-related and emotional experience.

### 1.2. School Streets as a Health-Oriented Urban Design Approach

Against this backdrop, School Streets have emerged as a targeted urban design and policy intervention to improve children’s safety, health, and everyday experiences around schools. School Streets typically involve restricting motorised traffic during school arrival and departure times, along with traffic-calming measures, anti-idling measures, and improvements to pedestrian and environmental quality. Evidence indicates that such interventions can reduce exposure to traffic-related risks, improve perceived safety, and support more active and socially rich daily routines for children [14,15,16,17,18].

Beyond measurable outcomes, such as traffic reduction or air quality improvements, School Streets transform the emotional experience of the school environment. By reducing traffic stressors and enhancing environmental comfort, these interventions can convert stress-dominated spaces into calmer, more legible, and socially supportive environments for children. In doing so, School Streets directly address many of the environmental determinants of children’s stress and comfort, including traffic exposure, crossing complexity, noise, and the absence of restorative features [19,20,21,22]. In addition, School Street interventions may contribute to more climate-responsive everyday environments by reducing traffic-related heat exposure and supporting the integration of shade, greenery, and more thermally comfortable pedestrian conditions around schools [23,24].

This is increasingly relevant in dense urban contexts, where the environmental quality of school surroundings is being reconsidered through the lens of urban greening, climate adaptation, and children’s well-being. Recent research has also highlighted inequalities in children’s exposure to green and blue infrastructure around schools in European cities, as well as the importance of greenery along home–school routes as part of everyday environmental experience [10,23,24].

Importantly, School Streets should be understood not only as transport measures but also as health-oriented spatial interventions. Their effectiveness depends not only on regulatory changes but also on how children experience the transformed environment. In contexts where School Streets are not yet institutionalised, child-centred empirical evidence is essential to inform context-sensitive design, prioritisation, and evaluation.

### 1.3. Why Children’s Experiential Evidence Matters

While research on active school travel and school-area environments has expanded significantly, much of the existing evidence remains focused on adult-defined indicators, such as infrastructure provision, traffic counts, and mode choice. Less attention has been paid to children’s emotional and experiential perspectives, despite growing recognition that children perceive and evaluate environments differently from adults.

Participatory and experiential research demonstrates that children attach strong emotional meaning to everyday spatial features that may appear minor from an adult’s planning perspective. Dark passages, obstructed sidewalks, visually chaotic intersections, and unfamiliar social situations can generate fear and stress, whereas greenery, landmarks, and quiet streets foster comfort and enjoyment [25,26,27,28]. Importantly, children may continue to use environments perceived as unsafe out of necessity, so exposure does not equate with comfort or well-being [19,25].

Recent studies conceptualise everyday routes as sequences of emotionally differentiated places, often described as “stress nodes” and “joy nodes.” Stress nodes—associated with traffic, noise, pollution, and spatial ambiguity—are linked to increased physiological stress responses and psychosomatic complaints, whereas joy nodes—characterised by greenery, calmness, and engaging features—support emotional regulation and motivation for active travel [1,8,12,29]. Participatory methods such as cognitive mapping, emotional mapping, and child-led documentation have proven effective in capturing these experiences and translating them into spatially explicit evidence relevant for urban design and public health. This makes children’s experiential evidence particularly valuable in contexts where formal School Street policies are absent, and design priorities must be identified through lived local experience.

### 1.4. Research Gap, Study Context, and Objectives

Belgrade provides a salient context for expanding the evidence base. While School Streets have been increasingly mainstreamed in many European cities, Serbia has not yet institutionalised this model as a distinct policy instrument. Nevertheless, several recent initiatives indicate an emerging alignment with its underlying principles, including active mobility and traffic safety programs [30,31] and municipal planning efforts that prioritise walking and cycling through Sustainable Urban Mobility Plans. Particularly relevant is the Handbook for Children- and Family-Friendly Design of Open Urban Spaces [32], developed with support from UNDP Serbia, which explicitly advocates safe, accessible, inclusive public spaces and participatory planning in which children and families act as co-creators—an agenda closely aligned with the School Street philosophy.

Against this backdrop, the present study explores how children perceive and emotionally experience their everyday routes to school in Belgrade and how these insights can inform health-oriented urban design strategies for School Streets. We address three research questions: (1) How do children perceive and emotionally experience streets and spaces along their daily school routes? (2) Which spatial features are associated with positive versus negative emotions, and how do these relate to established health determinants (safety, comfort, social cohesion, and restorative qualities)? (3) How can children’s experiential evidence support the context-sensitive design and implementation of School Streets in a city where the model is not yet formalised?

Accordingly, the objectives of this study are: (1) to explore how children perceive and emotionally experience their everyday routes to school; (2) to interpret these experiences through a health-oriented urban design lens; and (3) to examine how children’s experiential evidence can inform context-sensitive School Street design in Belgrade.

Conceptually, this study advances a health-oriented adaptation of the SCORELINE framework (h_SCORELINE) linking children’s participatory data to context-sensitive urban design criteria [33]. Methodologically, it positions children as capable evaluators and co-designers, whose emotional mappings can be systematically interpreted as design-relevant and policy-relevant evidence. This aligns with emerging international guidance on the creation of child-friendly public spaces [34,35] and the growing empirical literature evaluating the impact of school streets on active travel, satisfaction, and perceived safety [14,17,18].

By contributing rare child-centred evidence from a post-socialist urban context, this study strengthens international debates on healthy, child-friendly streets. It demonstrates how emotionally grounded participation can bridge public health goals with concrete, locally implementable street design interventions, rather than merely confirming that safety, greenery, and walkability matter. The study examines how children themselves spatially identify and emotionally prioritise these conditions in a context where School Streets have not yet been formalised.

## 2. Materials and Methods

This study adopted a child-centred, mixed-method participatory research design to explore how children perceive and emotionally experience their everyday routes to school and how these experiences relate to health-relevant qualities of the built environment. The methodological approach combines visual, tactile, and narrative tools appropriate for young children and is capable of capturing the emotional, spatial, and experiential dimensions of everyday mobility. The research was conducted over two consecutive school years with the same cohort of pupils, enabling a nuanced understanding of children’s perceptions at a critical stage of independent mobility development.

The methodological sequence was designed to combine context-sensitive participatory engagement with a later, more structured analysis of children’s spatial representations, allowing multiple forms of experiential evidence to be interpreted together.

### 2.1. Study Context, Participants, and Research Design

The study was conducted in Belgrade, Serbia, a post-socialist city characterised by rapid motorisation, traffic-dominated street environments, and limited implementation of child-oriented mobility interventions. While the concept of school streets is formally recognised in national and local regulatory frameworks, its practical application has not yet been implemented, and no known pilot projects or systematic research initiatives have been conducted to support their introduction in Belgrade. This gap between policy recognition and on-the-ground practice provides important context for the present study.

**Figure 1:**
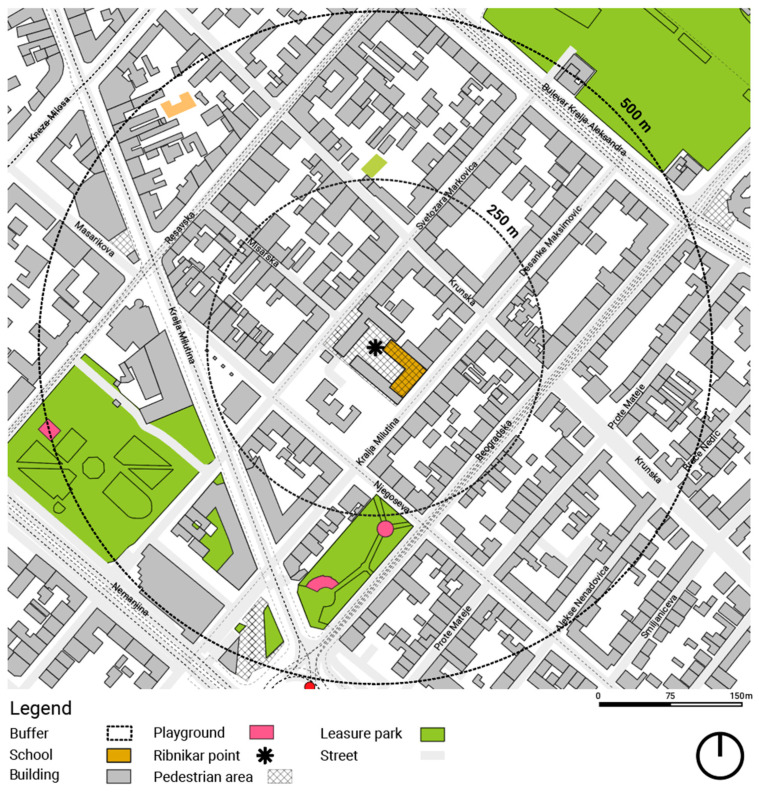
Location of the case-study school and its surrounding street environment. Source: M. Vukmirovic. Software: QGIS 3.40 (Bratislava) under the GNU General Public Licence, (QGIS Development Team, Open Source Geospatial Foundation, Beaverton, OR, USA).

The research was carried out at Model Primary School “Vladislav Ribnikar”, a public primary school located in a dense urban neighbourhood of central Belgrade (Figure 1). The surrounding street environment is characterised by mixed land use, high traffic volumes, on-street parking, and discontinuous pedestrian infrastructure—conditions that are typical of many school catchment areas in the city.

**Figure 2:**
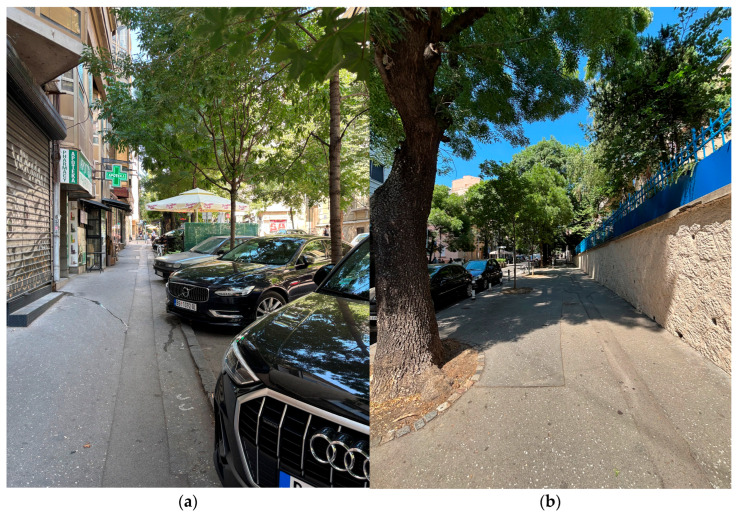
Selected street-level views of the school-route environment: (**a**) mixed-use street segment with narrow pedestrian space and curbside parking; (**b**) tree-lined sidewalk segment with greater shade, continuity, and pedestrian comfort. Source: M. Vukmirovic.

Selected street-level views of the school-route environment are presented in Figure 2, illustrating typical pedestrian conditions in the case-study area, including sidewalk continuity, curbside parking, active ground-floor uses, and the presence of greenery and shade.

Participants were pupils from one class who took part in two participatory workshops organised during a school day formally designated as “parent–teacher day” (dan “roditelj–učitelj”). This format, in which parents engage in educational activities alongside teachers, provides an appropriate and institutionally accepted framework for conducting participatory research with children.

The first workshop was conducted in October 2024 when the pupils were in the second grade (aged 7–8). The second workshop took place in November 2025 with the same cohort, who were then in the third grade (aged 8–9). The class consisted of 22 pupils. Working with the same group over two consecutive years ensured continuity of participation in the study. This continuity provided a richer basis for interpreting children’s spatial understanding and emotional articulation across two consecutive school years, at a stage of rapid cognitive and social development.

The study was designed as a two-stage, child-centred exploratory case study rather than as a longitudinal before-and-after intervention. The first workshop focused on children’s collective evaluation of the school street environment and route-related impressions. In contrast, the second introduced cognitive route mapping as a complementary method to deepen the understanding of spatial representation, route legibility, and emotionally meaningful locations.

### 2.2. Participatory Data Collection

Data collection combined three complementary participatory methods: (1) tactile and visual evaluation using Urban500 participatory boards, (2) cognitive route mapping through children’s drawings, and (3) photo documentation of everyday routes. These methods were supported by facilitated classroom discussion and interpreted as complementary sources of emotional, spatial, and experiential evidence.

#### 2.2.1. Urban500 Participatory Boards

During the first workshop (second grade), the children engaged with a set of Urban500 participatory boards adapted for use with young pupils. The boards were developed based on tools and principles derived from the National Association of City Transportation Officials (NACTO) guidelines, particularly those addressing the design of child-friendly streets, safe school environments, and public spaces prioritising pedestrians. These tools were translated and simplified to support the participatory evaluation and co-design of open public spaces from a child’s perspective.

The participatory boards employed visual symbols, pictograms, and clearly defined circular fields that corresponded to predefined response categories. Instead of using stickers or adhesive elements, the children were provided with coloured markers (felt-tip pens), which they used to mark and colour the circles directly on the posters. This approach was chosen to ensure simplicity, reduce distractions, and allow children greater freedom to express intensity and emphasis through colour choice and the marking style.

The structure of the boards reflects key dimensions commonly addressed in NACTO’s child-oriented street design approaches, such as safety, comfort, activity potential, and spatial quality, while being carefully adjusted to children’s cognitive and communicative abilities [35].

Children were asked to respond to prompts concerning emotions experienced in the street in front of their school, modes of travel to and from school, activities they would like to perform in the school-street environment, and physical elements they associated with a safe, pleasant, and child-friendly setting.

This was followed by a facilitated group discussion, during which the children explained their choices and elaborated on their drawings. This process generated both visual and narrative data, enabling the identification of shared perceptions, priorities, and concerns while grounding children’s input within an established urban design framework adapted for participatory use.

#### 2.2.2. Cognitive Route Mapping

In the second workshop (third grade), the children were asked to draw a map of their route from home to school. This cognitive mapping exercise builds on established approaches in urban studies and environmental psychology that use children’s drawings to reveal spatial understanding, perceived barriers, landmarks, and emotional associations [36].

Children were encouraged to depict their route freely and mark places they liked, disliked, or found frightening, as well as obstacles and points of interest. Emphasis was placed on experiential and emotional meaning rather than cartographic accuracy, allowing children to represent space according to personal significance, familiarity, and feelings.

During the activity, the children were invited to verbally explain their drawings, which helped clarify the emotional and spatial importance of specific locations. The resulting maps provide insights into children’s spatial legibility, perceived continuity and disruption along routes, emotional landmarks, and recurring patterns of perceived “stress” and “joy” nodes within their everyday mobility environments.

The mapping task did not aim to produce cartographically precise route data or a standardised mobility survey. Rather, it was used to capture how children selectively represent routes, landmarks, obstacles, and emotionally meaningful places within their everyday school-travel experience.

#### 2.2.3. Photo Documentation of Everyday Routes

Before the first workshop, the children were asked to take two photographs along their route to school: one representing a place or feature they liked and one representing a place or feature they disliked. Photographs were taken before the workshop session and submitted to the researcher in advance, enabling their incorporation into a PowerPoint presentation used during the class.

This child-led photo-documentation approach draws on photovoice methodologies and supports first-person perspectives on environmental quality. During the workshop, photographs were projected as visual prompts for guided discussion, with children invited to explain why particular places or features evoked positive or negative feelings along their route to school. This facilitated reflective conversations, enabling children to articulate their emotional responses, perceptions of safety, comfort, and attractiveness, and to link specific spatial characteristics to their everyday experiences.

The photographs were subsequently analysed alongside participatory board outputs and cognitive route drawings. Together, these materials provide concrete visual and narrative evidence of spatial features associated with comfort, fear, enjoyment, or discomfort, including traffic conditions, greenery, lighting, sidewalk quality, and spatial continuity. In this way, the photographs provided place-specific visual anchors for interpreting children’s route-related narratives and preferences.

#### 2.2.4. Facilitated Classroom Discussion

Facilitated classroom discussion was used throughout the research process as an interpretive component of the participatory design. During each activity, children were encouraged to explain their markings, drawings, and photographs through age-appropriate guided conversation, allowing the research process to move beyond visual output alone and to capture the meanings children attributed to particular places, spatial features, and everyday route experiences. These verbal explanations were especially important in clarifying why certain locations were associated with comfort, fear, enjoyment, stress, familiarity, or significance, and in identifying the experiential reasoning behind children’s spatial representations.

The discussions were not analysed as a separate, standalone dataset. Still, they were used to contextualise and support the interpretation of the three primary forms of material: participatory boards, cognitive route drawings, and photo documentation. In this way, facilitated discussion strengthened the internal coherence of the dataset and supported the triangulation of children’s emotional, spatial, and experiential accounts. It also enabled the identification of recurring themes that may not have been fully legible through drawings, photographs, or marked boards alone, particularly in relation to perceived safety, everyday obstacles, route preferences, and emotionally salient environmental features along the journey to school.

### 2.3. Ethical Considerations

The research design follows the principles of ethical, child-centred participatory research, recognising children as active contributors and experts in their everyday environments [34]. The study involved low-risk, non-invasive participatory activities conducted in a familiar educational setting. The activities were embedded within the school’s educational framework and conducted in cooperation with teachers and parents as part of the parent–teacher day program.

Participation was voluntary, and informed consent was obtained from parents or legal guardians before the research activities. The children were informed about the purpose of the workshops in age-appropriate language and were assured that there were no correct or incorrect answers. The activities were designed to be inclusive, non-evaluative, and playful, encouraging children to express their perceptions and emotions through drawing, poster marking, discussion, and photography.

All workshops were conducted in familiar classroom settings during regular school hours. No personally identifying data were collected, and all visual materials were anonymised before analysis. This study adhered to ethical standards for research involving children and aligned with international guidelines for participatory urban research with minors.

### 2.4. Analytical Strategy, Data Triangulation, and h_SCORELINE Interpretation

The qualitative analysis was structured around the SCORELINE framework, which conceptualises public space quality through a hierarchy of user needs, including safety and security, accessibility, legibility, comfort, inspiration, and liveability. In this study, SCORELINE was adapted into a health-oriented, child-centred analytical approach (h_SCORELINE) to examine how children emotionally and cognitively experience their everyday routes to school.

#### 2.4.1. Coding and Interpretation of Cognitive Maps

Children’s cognitive route drawings were treated as spatial–experiential data and systematically coded in NVivo 15 (Lumivero, 1331 17th Street, Suite 404, Denver, CO 80202, USA), using a codebook organised into four main groups: spatial elements, environmental qualities, drawing characteristics, and experiential and emotional cues. Within this mixed-method design, cognitive route drawings constituted the primary dataset for systematic relational analysis, while participatory boards, photographs, and workshop discussion provided complementary contextual material for interpretation.

To empirically operationalise the selected SCORELINE dimensions, a focused set of matrix-coding queries was applied. Rather than mirroring all SCORELINE criteria one-to-one, matrix queries were selectively developed for dimensions that could be examined directly through the relationships between spatial characteristics and experiential cues.

Four core matrix coding queries were conducted: (1) Safety and Security, examining the relationship between traffic-related spatial elements (e.g., intersections, transport infrastructure, barriers) and stress-related emotional cues; (2) Accessibility and Continuity, exploring how route structure, sidewalks, and obstacles relate to orientation, autonomy, and avoidance behaviours; (3) Legibility and Orientation, analysing associations between spatial clarity, landmarks, and children’s perceived orientation and sense of security; and (4) Comfort and Well-being, identifying positive associations between environmental qualities such as greenery, open space, and lighting and experiences of comfort, enjoyment, and safety. An additional optional matrix query examined drawing characteristics in relation to experiential cues to support the methodological interpretation of how children visually express stress and comfort.

Higher-order SCORELINE dimensions, such as inspiration and liveability, were not analysed through standalone matrix queries but were interpreted as integrative outcomes emerging from the combined effects of safety, accessibility, legibility, and comfort. Matrix coding queries thus functioned as an analytical mechanism that linked children’s lived spatial experiences with health-relevant dimensions of public space quality, enabling the identification of perceived stress and joy nodes along school routes and supporting the development of health-oriented urban design insights relevant to School Streets interventions. Because the study is exploratory and qualitative in character, the resulting counts, matrices, and heatmap visualisations are used descriptively to summarise patterns within the material rather than to support statistically generalisable claims.

#### 2.4.2. Cross-Reading of Boards and Photographs

Participatory boards and photo documentation were analysed through descriptive and thematic interpretation rather than through the NVivo coding procedure applied to the drawings. The boards provided aggregated insights into shared perceptions of the school-street environment, while the photographs helped identify place-specific likes, dislikes, and emotionally salient environmental features discussed by the children during the workshops.

#### 2.4.3. Triangulation Across Datasets

Triangulation was conducted by comparing recurring spatial, emotional, and experiential themes across the three main sources of material: cognitive route drawings, participatory boards, and child-led photographs, supported by verbal explanations generated during the workshops. The drawings provided the most structured insight into children’s representations of routes, landmarks, and emotionally salient locations. At the same time, the boards offered a more collective overview of perceived environmental qualities, everyday obstacles, and desired street elements. The photographs added place-specific visual anchors by capturing features that children explicitly identified as positive or negative along their routes to school. Spatial conditions, environmental features, or route segments that recurred across these sources were interpreted as more robust patterns within the case study. In this way, the analysis relied not on a single form of representation, but on the convergence of visual, spatial, and narrative evidence.

#### 2.4.4. Health-Oriented Reinterpretation Through h_SCORELINE

The triangulated findings were interpreted through h_SCORELINE as a health-oriented adaptation of the original SCORELINE framework, enabling children’s spatial and emotional experiences to be read in relation to everyday conditions relevant to well-being, comfort, safety, and independent mobility. In this way, recurring route features such as traffic exposure, obstructed sidewalks, poor lighting, greenery, recognisable landmarks, and wider pedestrian spaces were not considered only as physical attributes, but as environmental conditions associated with children’s reported or represented experiences of stress, insecurity, enjoyment, comfort, and orientation. This interpretive step made it possible to translate child-centred experiential evidence into a structured urban design reading relevant to the discussion of School Streets in Belgrade.

At the same time, the framework was applied cautiously and interpretively, without assuming that children’s emotions were produced solely by the built environment. Emotional responses during the journey to school may also be shaped by social interaction, routine, anticipation of school-related events, and other situational factors. Accordingly, h_SCORELINE was used not to establish direct causality, but to identify meaningful associations between spatial conditions and children’s lived everyday experiences, and to interpret these associations in a way relevant to health-oriented urban design.

The h_SCORELINE framework was operationalised by systematically linking core spatial elements, health-oriented interpretations, and empirical patterns identified in children’s participatory data. This relationship is synthesised in Figure 7, which presents the conceptual structure used to interpret the findings.

## 3. Results

### 3.1. Overview of Spatial–Emotional Patterns Across Participatory Materials

The analysis revealed consistent spatial–emotional patterns in children’s perceptions and experiences of their everyday mobility environments. These patterns were first identified in the immediate school-street context and were subsequently confirmed and extended through the analysis of children’s everyday routes. Together, they illustrate how specific spatial characteristics serve as recurring sources of emotional stress or comfort for children in their daily movements.

Across participatory activities, the children repeatedly associated certain spatial conditions with feelings of discomfort, fear, or insecurity. Traffic-dominated environments with obstructed or narrowed sidewalks, dark passages, and poorly maintained spaces emerged as key sources of negative emotional responses. These environments were commonly perceived as difficult to navigate independently and were often described or depicted as requiring heightened attention, caution, or adult supervision. Such spatial conditions formed clusters of perceived stress nodes that disrupted children’s sense of continuity, autonomy, and ease of movement.

Conversely, the children consistently identified spaces that supported positive emotional experiences. Green elements, including trees and small green pockets, and wider, visually legible sidewalks were associated with calmness, enjoyment, and a sense of safety. Culturally meaningful or recognisable places, such as well-known buildings or landmarks, also served as points of emotional reassurance, supporting children’s orientation and confidence in their routes. These locations functioned as perceived joy nodes, supporting emotional regulation and reinforcing positive walking experiences.

**Figure 3:**
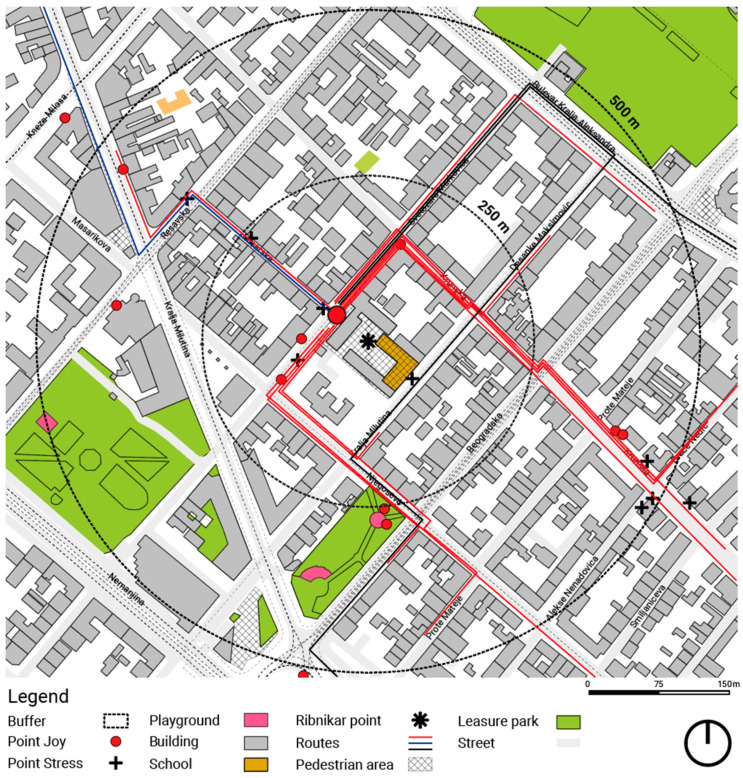
Spatial configuration of children’s school routes and emotional nodes in the study area. Source: M. Vukmirovic. Software: QGIS 3.40 (Bratislava) under the GNU General Public Licence.

The overall spatial distribution of children’s routes and the identified stress and joy nodes is shown in Figure 3, which provides a spatially grounded overview of the recurring emotional patterns discussed in the following subsections.

Spatial elements identified as emotionally significant in the school-front environment also reappeared along children’s wider everyday routes. In this sense, stressful and supportive conditions were not confined to the immediate school setting, but formed part of a broader experiential network shaping children’s daily mobility.

To support a health-oriented reading of the recurring spatial-emotional patterns identified across the participatory materials, Table 1 links children’s perceived spatial conditions with dominant emotional responses, their interpreted role within everyday routes, and their potential relevance for well-being and everyday mobility.

The relationships between spatial conditions, emotional responses, and health-oriented interpretations identified in the results are further synthesised in the conceptual diagram presented in Figure 7.

### 3.2. Structural Patterns in Children’s Cognitive Maps

The NVivo-based coding of children’s cognitive route drawings revealed a set of consistent structural patterns in the representation of space, movement, and experience (Table 2). Across all drawings (*n* = 18), the children demonstrated a strong reliance on recognisable spatial references and legible route structures, indicating that everyday mobility is primarily understood through concrete, experience-based elements rather than abstract spatial configurations.

#### 3.2.1. Modes of Spatial Representation

All children used landmarks as the primary organising elements of their cognitive maps (*n* = 18), indicating that spatial orientation was structured through identifiable points rather than through schematic street layouts. Orientation and legibility appeared in all drawings (*n* = 18), while most children emphasised the route itself (*n* = 15), suggesting that movement through space was more salient than static origin or destination points. Text labels were also frequent (*n* = 15), showing an effort to clarify spatial meaning, whereas scale distortion (*n* = 10) suggests that emotionally significant elements were often prioritised over proportional accuracy. Green emphasis appeared in several drawings (*n* = 8), typically in relation to positive spatial experience.

#### 3.2.2. Recognised Spatial Elements

Traffic-related components dominated children’s representations of their everyday environments. Transport infrastructure appeared in almost all drawings (*n* = 17), while streets and routes formed the basic structural framework (*n* = 16), alongside school and home as the two poles of the daily journey (*n* = 16 each). Intersections and crossings were also prominent (*n* = 14), often receiving heightened attention, while shops and services functioned as important orientation cues (*n* = 13). Physical barriers and obstacles were frequently noted (*n* = 10). In contrast, sidewalks and pedestrian paths appeared less often (*n* = 7), suggesting that pedestrian infrastructure was not consistently perceived as a distinct or secure spatial category.

#### 3.2.3. Perceived Environmental Qualities

Environmental qualities were represented selectively rather than systematically. Visual clarity was the most prominent attribute (*n* = 15), underlining the importance of legibility for children’s navigation and spatial confidence. Greenery and trees were present in over half of the drawings (*n* = 10), indicating that green elements formed a meaningful part of many children’s route experience. Maintenance and order (*n* = 6) and noise or disturbance (*n* = 6) became visible mainly when experienced as problematic, while openness appeared less frequently (*n* = 5). Lighting and darkness were only rarely depicted (*n* = 2), suggesting that not all experiential qualities were equally easy to visualise in drawing form.

#### 3.2.4. Experiential and Emotional Cues

The drawings suggest that emotional security was closely related to spatial comprehension. Orientation and legibility appeared in all drawings (*n* = 18), indicating that a sense of knowing where one is and how to proceed was a fundamental condition of everyday mobility. Comfort and pleasantness were expressed in several drawings (*n* = 8), while safety and security appeared selectively (*n* = 7), suggesting that these were context-dependent rather than taken for granted. Enjoyment and playfulness were linked to specific places (*n* = 6), and fear or stress also appeared in a notable share of the material (*n* = 6). By contrast, social presence was weakly represented (*n* = 3), and autonomy or independence was rarely expressed directly (*n* = 1), indicating that such dimensions may be only indirectly reflected in children’s spatial representations.

Taken together, these structural patterns suggest that children’s cognitive maps do not function merely as simplified route sketches, but as selective representations of spatially and emotionally meaningful everyday environments. The repeated emphasis on landmarks, crossings, barriers, greenery, and route continuity indicates that children’s drawings captured not only how they move through space, but also how they interpret its legibility, comfort, and potential sources of stress or ease.

### 3.3. Stress Nodes Along Children’s Everyday Routes

**Figure 4:**
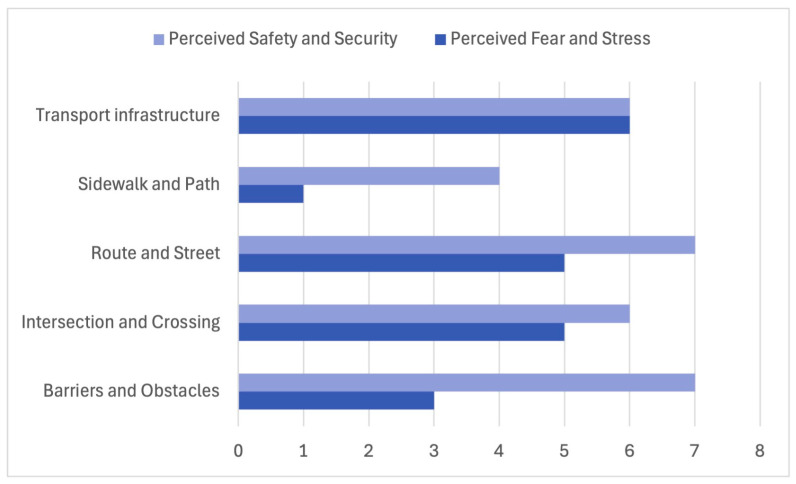
Spatial configurations associated with perceived fear, stress, and safety.

Building on the structural patterns identified in the NVivo coding of cognitive maps (Table 2), the matrix coding analysis revealed clear spatial configurations associated with perceived fear, stress, and safety (Figure 4). Consistent with the frequent representation of traffic-related elements in children’s drawings, traffic-dominated spatial components emerged as the primary stress points along school routes. Transport infrastructure showed the highest co-occurrence with perceived fear and stress (*n* = 6), followed by intersections and crossings (*n* = 5), and main routes and streets (*n* = 5). These same elements are also strongly associated with children’s awareness of safety and security, indicating that locations perceived as potentially dangerous were also associated with heightened attention and caution.

Barriers and obstacles constitute another important category of stress-related factors. Although they are depicted less frequently than traffic infrastructure, they combine moderate levels of fear (*n* = 3) with a high frequency of safety-related codes (*n* = 7). This pattern suggests that physical discontinuities, such as parked cars, narrow passages, or obstructed sidewalks, are cognitively salient and clearly identified as problematic, even when fear is not always explicitly expressed. In contrast, sidewalks and pedestrian paths exhibited the lowest association with fear and stress (*n* = 1), reinforcing their role as emotionally safer components of everyday mobility environments.

Overall, these results indicate that children’s perceived stress is concentrated in traffic-dominated, spatially complex locations, corroborating the prominence of transport infrastructure, intersections, and major streets identified in the initial NVivo analysis of spatial elements. These locations therefore emerge as priority points for safety-oriented interventions along school routes.

### 3.4. Joy Nodes, Comfort, and Emotional Well-Being

**Figure 5:**
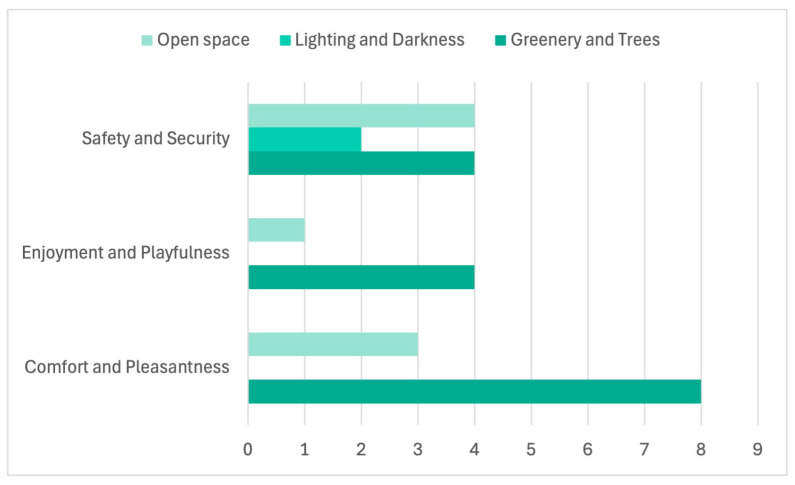
Spatial conditions associated with comfort, enjoyment, and emotional well-being.

In contrast to stress-related patterns, the analysis of environmental qualities in relation to experiential cues highlights a distinct set of spatial conditions associated with comfort, enjoyment, and emotional well-being (Figure 5). Consistent with the frequent visual emphasis on greenery identified in the structural coding of drawings (Table 2), greenery and trees emerged as the most significant contributors to children’s sense of comfort and pleasantness (*n* = 8). Green elements are also associated with enjoyment and playfulness (*n* = 4) and perceived safety (*n* = 4), underscoring their role as positive emotional anchors in children’s everyday mobility environments.

Open spaces showed a moderate association with comfort (*n* = 3) and safety (*n* = 4), but only a limited link to enjoyment and playfulness (*n* = 1). This suggests that spatial openness alone does not guarantee positive emotional engagement, particularly in the absence of complementary qualities, such as greenery or activity-supporting features. In contrast, light and darkness were not associated with comfort or enjoyment. Still, they were related exclusively to perceived safety (*n* = 2), indicating that children interpret light primarily as a protective rather than an experiential feature of the environment.

Together, these findings indicate that joy nodes along school routes are closely tied to environmental qualities that support sensory comfort, emotional regulation, and positive route experience, with greenery functioning as a particularly salient feature in children’s representations.

### 3.5. Legibility, Accessibility, and Continuity in Children’s Route Experience

Legibility-related features constitute one of the most consistent and robust patterns across all stages of the analysis. As already evident in the universal emphasis on landmarks and orientation cues in children’s cognitive maps (Table 2), the matrix coding results further confirm that children overwhelmingly express spatial orientation through recognisable reference points. Landmark emphasis showed the highest frequency of co-occurrence with orientation and legibility codes (*n* = 37), followed by text labels (*n* = 24) and visual clarity (*n* = 26).

**Figure 6:**
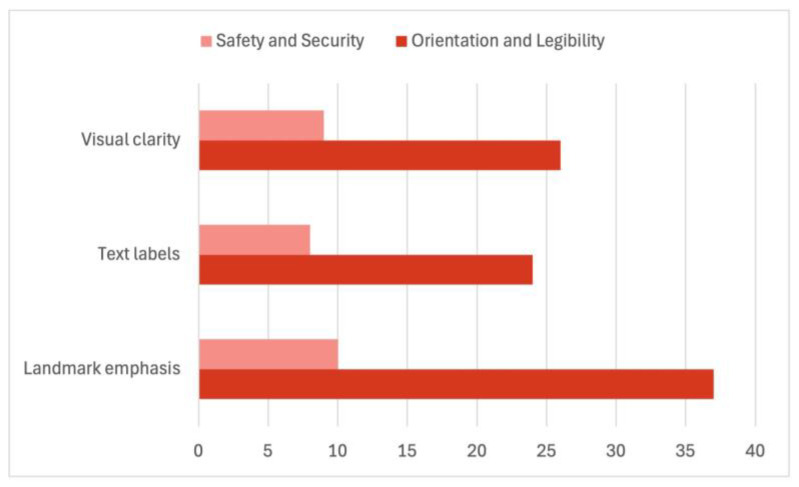
Legibility and Orientation as Health-Supportive Spatial Factors.

Notably, all three legibility-related features were strongly associated with perceived safety and security. Landmark emphasis (*n* = 10), visual clarity (*n* = 9), and text labels (*n* = 8) consistently co-occurred with safety-related coding, indicating that spatial comprehensibility functions as a health-supportive factor by reducing uncertainty and emotional stress (Figure 6). These findings reinforce the interpretation that children’s emotional security is closely linked to their ability to read and interpret the environment through familiar, meaningful cues rather than through abstract spatial structures.

Children’s representations further suggest their awareness of how spatial design influences their independence. Visually clear, predictable, and less traffic-conflicted environments were more frequently associated with confidence and willingness to walk independently. In contrast, spaces characterised by ambiguity, visual clutter, or vehicular dominance are linked to hesitation and discomfort. These findings suggest that children’s emotional responses are closely tied to their assessment of spatial affordances and constraints.

Patterns related to accessibility and continuity reveal important underlying spatial conditions that shape children’s everyday mobility experiences. While these features are less directly associated with emotional outcomes, they play a critical role in structuring children’s ability to navigate and understand their surroundings. Barriers and obstacles do not co-occur with autonomy or avoidance. However, they are strongly associated with orientation and legibility (*n* = 11), indicating that discontinuities are cognitively salient even when they are not explicitly articulated as avoidance behaviour.

Routes and streets (*n* = 23) and sidewalks and paths (*n* = 13) were also strongly associated with orientation and legibility, suggesting that continuous and readable pedestrian infrastructure supports children’s capacity to navigate independently. Explicit expressions of autonomy and independence were rare (*n* = 1), and avoidance behaviours were not depicted directly, suggesting that constraints on independent mobility may be internalised or expressed indirectly through spatial representations rather than through overt behavioural strategies.

Across the analyses, a coherent pattern emerged in which traffic-dominated and spatially discontinuous environments functioned as stressors, whereas greenery, spatial legibility, and environmental clarity supported comfort, orientation, and emotional security. The initial coding of cognitive maps provided a structural basis for these relational findings by showing how children’s modes of spatial representation aligned with health-relevant experiential patterns.

## 4. Discussion

### 4.1. Children’s Emotional-Spatial Evidence as a Health-Relevant Layer of Urban Design Knowledge

This study shows that children’s emotional experiences of daily school routes constitute a health-relevant link between urban design and public health. These associations should be interpreted cautiously, as children’s emotional responses during the journey to school may also be shaped by routine, social interaction, and anticipation of school-related events, rather than by spatial conditions alone. When analysed through the h_SCORELINE framework, children’s cognitive route representations reveal that stress, comfort, and enjoyment are tied to specific spatial conditions. These results reinforce that children’s emotional perceptions mediate the link between their environment, active mobility, and health [1,2,3,4,5].

The results show that children’s emotional-spatial patterns align closely with a health-oriented urban design reading. Recurring stress and joy points across the school and routes highlight the impact of daily spatial exposure. The spatial configuration shown in Figure 1 further illustrates how emotional experiences are not randomly distributed, but are associated with specific urban conditions along children’s everyday routes. These emotional geographies reveal how urban streets can support or hinder well-being, especially with active mobility. Children’s emotional responses are spatially patterned, forming stress and joy nodes along routes. This confirms emotional experiences are a vital aspect of health-oriented urban design, alongside traditional infrastructure and traffic metrics [7,29].

**Figure 7:**
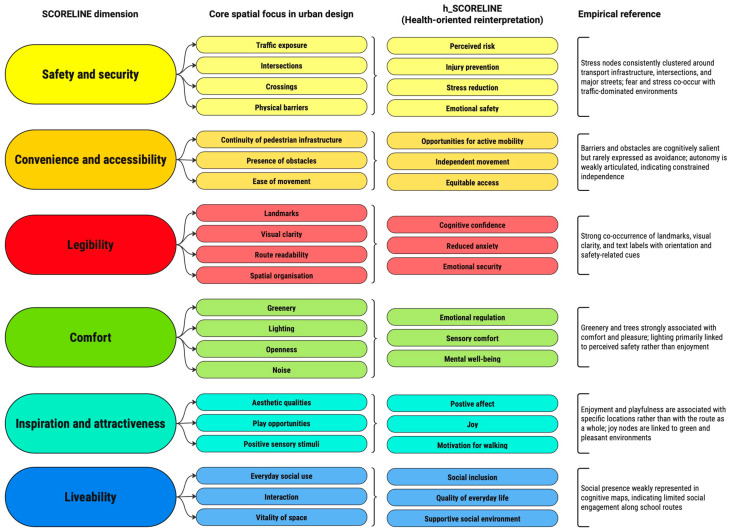
Operationalisation of the h_SCORELINE framework linking spatial conditions, health-oriented interpretations, and children’s experiential evidence.

This study uses the h_SCORELINE framework to connect children’s experiential data with urban design principles, reinterpreting SCORELINE’s key dimensions through a public health lens. Figure 7 illustrates this conceptual transition by showing how the original SCORELINE dimensions are reinterpreted through a health-oriented lens and linked to the empirical patterns identified in children’s cognitive maps. This approach links children’s emotional experiences to health-related spatial conditions, facilitating discussion of evidence-based, child-centred urban design.

In practical terms, these relationships can inform context-sensitive urban design interventions. For example, spatial configurations associated with perceived risk and stress highlight the need for traffic-calming and safer crossings; discontinuities in pedestrian infrastructure underscore the importance of continuous, unobstructed sidewalks; and the strong association between greenery and positive emotional responses supports the integration of shade and vegetation along school routes. In this way, children’s emotional-spatial evidence can complement conventional traffic and environmental metrics in guiding the implementation of School Streets.

Identifying stress and joy nodes in children’s cognitive maps shows emotional geographies as a health-relevant layer. Stress nodes linked to traffic, intersections, and fragmented infrastructure; joy nodes near green areas, clear routes, and quieter streets. These findings support research connecting children’s emotional perceptions—fear, comfort, and enjoyment—to active school travel and well-being [1,2,37]. Using the h_SCORELINE framework, the study reveals that children’s emotional responses to environments are internalised rather than merely inferred from adult perceptions, highlighting emotions as key to how spatial conditions affect health and behaviour. The contribution of the study, therefore, lies not in restating that safety, greenery, or walkability matter in general, but in showing how children themselves spatially identify and emotionally prioritise these conditions in a context where School Streets have not yet been formalised.

### 4.2. Interpreting Key Spatial Determinants of Everyday Well-Being

#### 4.2.1. Traffic Exposure and Stress

The concentration of stress nodes around traffic-dominated streets and intersections reinforces extensive evidence linking traffic exposure to perceived risk, fear, and reduced likelihood of walking or cycling to school [19,20,21,38]. While much of the existing literature relies on parental perceptions or objective indicators, the present study adds a child-centred experiential dimension by showing how traffic conditions are emotionally encoded in children’s spatial representations [8,12]. At the same time, not all heightened alertness in traffic environments should be interpreted as uniformly negative, since some degree of vigilance may also reflect adaptive learning in navigating urban space. However, repeated exposure to places associated with fear, uncertainty, or discomfort remains relevant from a health-oriented design perspective.

From a health-oriented urban design perspective, this is particularly relevant because the identified stress nodes correspond to spatial conditions that can be modified through design and policy interventions. Traffic calming, restricted vehicle access, simplified crossings, and reduced curbside obstructions can therefore be understood not only as safety measures but also as interventions that reduce chronic emotional stress associated with daily mobility [14,15]. These findings support the framing of everyday school routes as preventive health environments rather than merely transport corridors.

#### 4.2.2. Greenery, Comfort, and Emotional Regulation

The strong association between greenery and positive emotional experiences underscores the importance of daily exposure to natural elements for children’s well-being. Consistent with previous findings, exposure to vegetation and visually calm environments is associated with comfort, enjoyment, and reduced stress, even when such elements are embedded in traffic-dominated routes [7,10,11].

Children’s selective emphasis on greenery in their drawings suggests that green elements serve as emotional anchors within everyday mobility environments. This supports the argument that health-supportive qualities need not be confined to parks or large green spaces but can be effectively integrated into routine streetscape design. From a design perspective, these findings strengthen the case for small-scale, distributed green interventions, such as street trees, planted buffers, and shaded sidewalks, as part of School Street and active mobility strategies. Although the present study focused primarily on route environments rather than on a systematic assessment of school grounds, the findings also suggest the need for future research comparing route-related greenery with the presence or absence of natural elements within the school setting itself.

#### 4.2.3. Legibility and Emotional Security

Legibility emerged as a consistent protective factor in children’s cognitive maps. Reliance on landmarks, visual clarity, and readable routes indicates that spatial comprehensibility plays a central role in children’s emotional security. Locations perceived as legible were more frequently associated with feelings of safety and comfort, whereas visually chaotic and ambiguous environments were associated with stress and uncertainty. The strong role of landmarks, including the school itself, may be partly expected given the destination-oriented nature of the journey. Still, it nevertheless confirms the importance of legibility and recognisable reference points in children’s everyday spatial security.

These findings align with recent research on the built environment, which demonstrates that visual coherence and route clarity contribute to comfort and active mobility, particularly among children [11,39]. From a health perspective, improved legibility may reduce cognitive load and emotional strain, supporting children’s confidence and willingness to move independently through everyday environments [9]. Because route lengths, accompaniment patterns, and travel modes were not systematically standardised in this study, these findings should be read as experiential and interpretive rather than as a full behavioural account of independent school mobility.

#### 4.2.4. Accessibility, Continuity, and Constrained Autonomy

Although explicit expressions of autonomy were limited in the children’s drawings, the analysis revealed how spatial barriers and discontinuities may silently constrain independent mobility. Barriers and obstacles were cognitively salient and closely linked to orientation challenges, even when avoidance behaviours were not explicitly depicted. This finding aligns with evidence that children may adapt to spatial constraints without articulating them, normalising limited independence over time [3,4,5].

From a public health perspective, the normalisation of constrained autonomy has important implications. Reduced opportunities for independent mobility are associated with lower physical activity levels and diminished emotional well-being, reinforcing the importance of accessibility and pedestrian continuity as foundational conditions for healthy mobilisation [1,7].

### 4.3. From Child-Centred Evidence to Context-Sensitive School Street Design in Belgrade

In cities such as Belgrade, where School Street policies have not yet been institutionalised, the findings of this study provide an evidence-based rationale for pilot interventions. In practical terms, the findings point to a set of locally relevant priorities, including traffic calming near school entrances, simplified and safer crossings, removal of curbside obstructions, protection of sidewalk continuity, additional shade and greenery, and low-cost pilot interventions that improve children’s sense of comfort and orientation along the school route. School Streets directly address the spatial conditions identified as stress nodes—traffic exposure, complex crossings, and environmental discontinuities—while creating opportunities to enhance legibility, comfort, and emotional security. At the same time, these implications should be understood in relation to the specific context of a centrally located Belgrade school. They may differ in peripheral or socioeconomically less advantaged neighbourhoods, where children may face other dominant spatial stressors.

Importantly, School Streets emerged here not as a predefined solution but as a spatial response aligned with children’s experienced stressors and everyday emotional realities. The study demonstrates that children’s emotional experiences constitute legitimate and policy-relevant evidence that can inform the prioritisation, design, and evaluation of School Street initiatives. In contexts where quantitative health or mobility data may be limited, child-centred experiential evidence offers a valuable complement to health-oriented urban design and planning. In this sense, child-centred emotional-spatial evidence can function not only as a descriptive supplement to conventional planning data but as a context-sensitive basis for identifying priority intervention points in the gradual implementation of School Streets.

## 5. Conclusions

This study shows that children’s everyday school routes can provide important experiential evidence for health-oriented urban design. Across the participatory materials, children consistently associated traffic-dominated streets, complex crossings, and interrupted pedestrian continuity with discomfort, fear, and insecurity, while greenery, spatial legibility, and visually calm environments were associated with comfort, enjoyment, and emotional ease. These findings suggest that children’s route experiences can help identify recurring stress nodes and joy nodes within the everyday mobility environment.

Methodologically, the study demonstrates the value of combining participatory boards, cognitive route drawings, photo documentation, and facilitated discussion to interpret children’s spatial experiences through a health-oriented lens. In this context, the h_SCORELINE functioned as an interpretive framework that linked children’s emotional and spatial evidence to urban design qualities relevant to safety, comfort, orientation, continuity, and well-being. The findings further suggest that limited expressions of autonomy in children’s representations may reflect not an absence of concern, but the everyday normalisation of constrained mobility conditions.

For Belgrade, the study supports the gradual introduction of School Streets as a context-sensitive strategy for improving children’s daily environments. The results point to concrete priorities, such as traffic-calming near school entrances, safer, simpler crossings, improved sidewalk continuity, reduced curbside obstruction, and the strengthening of greenery and shade along school routes. More broadly, the study highlights that the evaluation of such interventions should not rely solely on traffic or environmental indicators but should also consider children’s emotional experiences of space as a meaningful form of evidence in shaping healthier, more inclusive street environments. In this sense, children should be understood not as passive users of school-route environments, but as knowledgeable participants whose everyday experiences can help guide healthier urban design decisions.

The proposed h_SCORELINE model provides a transferable framework for integrating children’s emotional experiences into health-oriented urban design and identifying context-specific priorities for interventions such as School Streets.

## 6. Limitations and Future Research

This study has several limitations that should be considered when interpreting its findings. First, it is based on a relatively small sample from a single school in central Belgrade, which represents a specific socio-spatial context and may reflect more favourable urban and socioeconomic conditions than those found in peripheral or less resourced neighbourhoods. For this reason, the findings should be understood as context-sensitive rather than statistically representative, and the dominant stressors identified here may differ from those shaping children’s everyday mobility in other urban settings.

Second, the study relied on participatory, perceptual, and representational forms of evidence, including cognitive route drawings, photo documentation, and classroom-based interpretation. These methods are well-suited to capturing lived experience, but they also involve an interpretive dimension shaped by children’s expressive abilities, workshop dynamics, and the research context itself. In addition, route lengths, accompaniment patterns, and travel modes were not systematically standardised, meaning that the findings should be read as experiential and interpretive rather than as a full behavioural account of school mobility. The results, therefore, reflect perceived and expressed experiences, rather than direct physiological, clinical, or travel-behaviour outcomes.

Third, the study focused specifically on the journey to school and its immediate street environment. While this focus is highly relevant to the discussion of School Streets, it does not capture the full emotional geography of childhood in the city. Other spaces, such as parks, playgrounds, recreational areas, and informal peer environments, may also play an important role in shaping children’s emotional relationships with urban space. Similarly, the present study did not systematically compare greenery along school routes with the presence or absence of natural elements within the school grounds, an important direction for future research.

Finally, the cross-sectional design did not allow for the assessment of change over time or for before-and-after comparison. Future studies could strengthen the evidence base by combining participatory methods with longitudinal designs, comparative neighbourhood case studies, route-tracking or mobility survey data, objective environmental measurements, and evaluations of School Street interventions before and after implementation.

At the same time, these limitations underscore the broader value of the study. The case-specific and child-centred nature of the research enabled the generation of detailed experiential evidence that is often absent from conventional planning and mobility assessments. In this sense, the methodological approach and analytical framework offer a useful foundation for future research and practice seeking to integrate children’s emotional experiences into health-oriented urban design and policy.

## Figures and Tables

**Table 1:** Translating children’s spatial–emotional experiences into health-oriented urban design insight.

Observed Spatial Condition (Children’s Perspective)	Dominant Emotional Response	Interpreted Spatial Role	Health-Oriented Relevance
Traffic-dominated streets and crossings	Fear, insecurity	Stress node	Elevated psychological stress; reduced willingness for independent mobility
Obstructed or narrow sidewalks	Discomfort, frustration	Stress node	Perceived loss of control; barriers to active mobility
Dark passages and visually enclosed spaces	Anxiety, avoidance	Stress node	Increased perceived risk; negative emotional regulation
Green elements and tree-lined sidewalks	Calmness, enjoyment	Joy node	Restorative exposure; support for emotional well-being
Wide, legible walking paths	Confidence, ease	Joy node	Cognitive comfort; support for autonomy
Recognisable landmarks and active frontages	Orientation, reassurance	Joy node	Emotional stability; sense of belonging

**Table 2:** Analysis of cognitive maps: spatial elements, qualitative characteristics, and emotional indicators (*n* = 18).

Code	Files (*n*)	Interpretation
**A. Drawing characteristics (modes of spatial representation)**		
Landmark emphasis	18	All children use recognisable points to structure their routes; space is understood through landmarks rather than abstract maps
Route emphasis	15	Most drawings emphasise the line of movement rather than the origin or destination points
Text labels	15	Children use written labels to clarify spatial meaning, indicating conscious spatial mapping
Scale distortion	10	Frequent disproportions indicate the emotional significance of specific elements
Green emphasis	8	Greenery is visually amplified when it carries positive value
Repetition	4	Repetition appears selectively, primarily in relation to salient elements
**B. Spatial elements (what children recognise in space)**		
Transport infrastructure	17	Traffic-related elements are an almost universal component of children’s spatial experience
Route and Street	16	The street represents the basic structure of everyday mobility
School	16	The school is a strong spatial reference, often clearly marked
Home	16	Home and school define the two poles of the route
Intersection and Crossing	14	Intersections function as critical points along the route
Landmarks—shops and services	13	Commercial uses serve as key orientation points
Barriers and Obstacles	10	Physical barriers are a frequent component of children’s experience
Sidewalk and Path	7	Sidewalks are not universally recognised as safe or prominent spaces
Playgrounds and Play Space	6	Play is present but secondary to movement-related functions
Open Space	5	Open spaces appear selectively in children’s representations
Landmarks—cultural and religious	3	Cultural and religious landmarks play a limited role
**C. Environmental qualities (what the space is like)**		
Visual clarity	15	Spatial legibility is a key qualitative attribute
Greenery and Trees	10	Green elements are present in most, but not all, drawings
Maintenance and Order	6	Noted primarily when disrupted or particularly pronounced
Noise and Disturbance	6	Noise emerges as a relevant stressor
Spatial openness	5	Openness is perceived as a positive spatial quality
Traffic presence	3	Traffic is more often implied than explicitly drawn
Lighting and Darkness	2	Darkness is rarely visualised explicitly
Spatial narrowness	0	Spatial constriction is not recognised as a distinct concept
**D. Experiential and emotional cues (how children feel)**		
Orientation and Legibility	18	Emotional security is directly linked to spatial legibility
Comfort and Pleasantness	8	Feelings of comfort are expressed by nearly half of the children
Safety and Security	7	Safety is not taken for granted but selectively perceived
Enjoyment and Playfulness	6	Positive emotions are associated with specific locations
Fear and Stress	6	Stress is present in approximately one-third of the pupils
Social presence	3	Social interactions are weakly represented
Autonomy and Independence	1	Independence is rarely expressed explicitly
Avoidance and Detour	0	Avoidant behaviours are implicit rather than directly depicted

## Data Availability

The data presented in this study are not publicly available due to ethical and privacy restrictions related to research involving children. Anonymised qualitative data supporting the findings may be made available by the corresponding author upon reasonable request.
